# Meta-analysis on the safety and efficacy of different dressings in the treatment of diabetic foot ulcers

**DOI:** 10.1515/med-2026-1459

**Published:** 2026-06-26

**Authors:** Xinxin Huang, Xubiao Meng

**Affiliations:** Haikou Hospital Affiliated of Xiangya School of Medicine, Central South University, Haikou, China

**Keywords:** dressings, diabetic foot ulcer (DFU), healing, wound infection, ulcer area

## Abstract

**Introduction:**

Diabetic foot ulcers (DFU) are a common and severe complication of diabetes, associated with high morbidity, infection risk, and amputation rates. Various dressings have been applied in clinical practice, yet their comparative safety and efficacy remain unclear. This study aimed to systematically evaluate different dressings for DFU treatment through meta-analysis.

**Content:**

Relevant studies from Chinese and English databases were retrieved and analyzed. A total of 25 studies involving 2,421 patients were included. Dichotomous data analysis showed that the experimental group had a significantly higher complete wound healing rate than the control group (OR=4.62, 95 % CI: 3.10–6.90, p<0.01), a significantly lower incidence of wound infection (OR=0.63, 95 % CI: 0.43–0.93, p=0.02), and no significant difference in amputation rate (OR=0.62, 95 % CI: 0.34–1.14, p=0.12). Continuous data analysis indicated that the experimental group had higher wound healing scores (MD=25.77, 95 % CI: 2.33–49.20, p=0.03), greater ulcer area reduction (MD=27.96, 95 % CI: 21.93–34.00, p<0.01), and shorter healing time (MD=–11.41, 95 % CI: –14.35 to –8.47, p<0.01). Meta-regression analysis suggested that publication year, blinding, and outcome indicators contributed to heterogeneity (p<0.05).

**Summary:**

Activated carbon cloth (ACC), negative pressure wound therapy (NPWT), and olive oil dressings demonstrated favorable safety and efficacy, although each has distinct advantages. Clinical selection should therefore be individualized according to patient conditions and wound characteristics.

**Outlook:**

Future studies with larger sample sizes and higher methodological quality are needed to further validate these findings and reduce heterogeneity, thereby providing more robust evidence for clinical decision-making.

## Introduction

Diabetic foot (DF) is a complication of diabetes caused by poor glycemic control, leading to vascular and neurological damage as well as local muscle atrophy [1]. Prolonged hyperglycemia results in persistent arterial sclerosis and lipid-glucose metabolic disorders, which contribute to inadequate blood supply to the feet, reduced pain sensation, and numbness. Patients are also highly susceptible to bacterial and fungal infections, and even minor injuries may deteriorate rapidly, ultimately developing into diabetic foot ulcers (DFU) [2], 3]. DFUs usually occur on the plantar surface or between the toes, and are often accompanied by pus, foul odor, redness, and swelling. In severe cases, gangrene, osteomyelitis, sepsis, and even amputation or life-threatening conditions may occur [4]. Therefore, timely debridement of necrotic tissue, combined with the application of dressings to promote granulation tissue growth and wound healing, is crucial for restoring or preserving sensory and motor functions of the affected foot. Traditional dressings such as oil gauze and herbal preparations can effectively maintain moisture, reduce adhesion between the dressing and wound surface, and prevent secondary injury [5]. Functional dressings, including gels and membranes, help relieve discomfort such as redness and burning pain, while partially repairing wound cavities or sinus tracts, thus improving patient comfort [6]. Moreover, depending on the size and depth of the ulcer, antimicrobial, composite, and bioactive dressings also provide significant advantages in DFU treatment [7], 8]. However, given the wide variety of dressings available and the frequent coexistence of multiple complications in patients with diabetic foot, the clinical situation is highly complex, and no consensus has been reached regarding the most suitable dressing for post-debridement DFU management. Therefore, this study conducted a meta-analysis of different dressings for the treatment of DFU, aiming to identify the treatment strategies with the greatest clinical value, thereby improving therapeutic outcomes and restoring foot health.

## Data sources and methods

### Data sources

Literature related to “the safety and efficacy of different dressings for DFU” was searched in both Chinese and English databases, covering the years 2000–2025. In Chinese databases – Wanfang Med, China National Knowledge Infrastructure (CNKI), X-MOL, and Google Scholar – the Chinese search terms included: “diabetic foot ulcer,” “silver dressings,” “moist dressings,” “hydrogels,” “oil-based dressings,” “aloe gel,” and “biological dressings.” In English databases – Wiley InterScience, PubMed, Web of Science, Cochrane Library, and SpringerLink – the search terms included: “DFU,” “silver dressings,” “sucrose octasulfate dressing,” “biological dressing,” “hydrogels,” and “different dressings.”

### Literature screening

#### Inclusion criteria

① Studies published between 1990 and 2025, with written informed consent obtained from all participants. ② Studies closely related to “the safety and efficacy of different dressings in the treatment of DFU”. ③ Participants were patients with foot ulcers caused by long-term progression of diabetes. ④ Patients had no history of amputation, malignant tumors, congenital immune disorders, other endocrine or metabolic diseases, or severe hepatic/renal dysfunction. ⑤ Patients had no foot trauma, fractures, deformities, or other dermatological diseases.

#### Exclusion criteria

① Unpublished literature, journal-retracted articles, or studies involving academic copyright disputes. ② Literature with incomplete information (e.g., unknown authors, missing volume/issue/page numbers, lack of abstract, unobtainable full text, unclear research methods/objectives, or missing data). ③ Duplicate publications. ④ Studies with obvious errors (e.g., mistakes in experimental procedures, scoring criteria, or observation duration). ⑤ Articles categorized as systematic reviews, quantitative analyses, network pharmacology studies, narrative reviews, conference proceedings, other meta-analyses, animal experiments, survey reports, or case reports. ⑥ Low-quality studies (defined as having four or more domains rated as “high risk” in the seven-item risk-of-bias assessment), deemed to have limited research value.

### Outcome measures

① Complete wound healing rate. ② Adverse events. ③ Recurrence. ④ Wound healing score. ⑤ Ulcer depth change. ⑥ Ulcer area change. ⑦ Laboratory indicators. ⑧ Blood perfusion of the affected foot. ⑨ Microbial distribution. ⑩ Healing time. ⑪ Pain score. ⑫ Home care duration. ⑬ Quality of life.

### Literature screening and data extraction

Literature screening and data extraction were performed in strict accordance with the PRISMA framework, including identification, screening, eligibility, and inclusion. After retrieving relevant literature from Chinese and English databases using keywords, all titles were imported into the NoteExpress V4.X for duplication checking. Following de-duplication, titles and abstracts were carefully screened to exclude low-quality and poorly relevant studies. Two investigators then independently screened the records according to the inclusion and exclusion criteria and extracted data for summary. Any disagreements were resolved through adjudication by a third reviewer with richer clinical experience and higher professional title. Data extracted included: ① Basic bibliographic information: first author, year of publication, journal, and country. ② Participant characteristics: source of participants, sex, age, number of cases completing the study, ulcer type and area, dressing material, and treatment duration. ③ Study characteristics: design, methods, objectives, content, outcome measures, and results. ④ Key factors relevant to the risk-of-bias assessment.

### Risk of bias assessment

The quality of the included studies was assessed using the Cochrane Risk of Bias Tool in the web-based Review Manager (RevMan) 5.4. The assessment covered the following domains: ① Randomization methods, such as random sequence generation, grouping by disease type, admission time, or overall randomization, which may introduce selection bias. ② Allocation concealment, which may introduce selection bias. ③ Blinding of participants and personnel, which may introduce performance bias. ④ Blinding of outcome assessment, which may introduce detection bias. ⑤ Completeness of outcome data, which may introduce attrition bias. ⑥ Selective outcome reporting, which may introduce reporting bias. ⑦ Other potential sources of bias, such as studies targeting specific populations or possible fabrication of research findings. Each of the seven domains was rated as “low risk,” “high risk,” or “unclear risk.” The summarized results were then visualized using the built-in “Figure” function of RevMan 5.4 to generate risk-of-bias plots.

### Statistical analysis

Data were analyzed using the meta-analysis module in Stata 18.0. For dichotomous variables, effect sizes were expressed as odds ratios (OR) with 95 % confidence intervals (CI). For continuous variables, mean differences (MD) with 95 % CI were calculated when measurement units were consistent; otherwise, standardized mean differences (SMD) with 95 % CI were used. Heterogeneity among studies was assessed using the Q test and quantified with the I^2^ statistic. If no significant heterogeneity was observed (p>0.1, I^2^≤50 %), a fixed-effect model was used to calculate pooled OR/MD/SMD with 95 % CI and to generate forest plots. If significant heterogeneity was present (p≤0.1, I^2^>50 %), a random-effects model was applied for pooled effect estimates and forest plot generation. Z-tests were conducted for pooled OR values and 95 % CI, with p<0.05 considered statistically significant. Publication bias was assessed and funnel plots were generated for the outcome with the largest number of included studies. After coding or transforming all study data, meta-regression analyses were performed to explore the presence and potential sources of heterogeneity.

### Research ethics

Not applicable.

### Informed consent

Not applicable.

## Results

### Literature screening results

**Figure 1:**
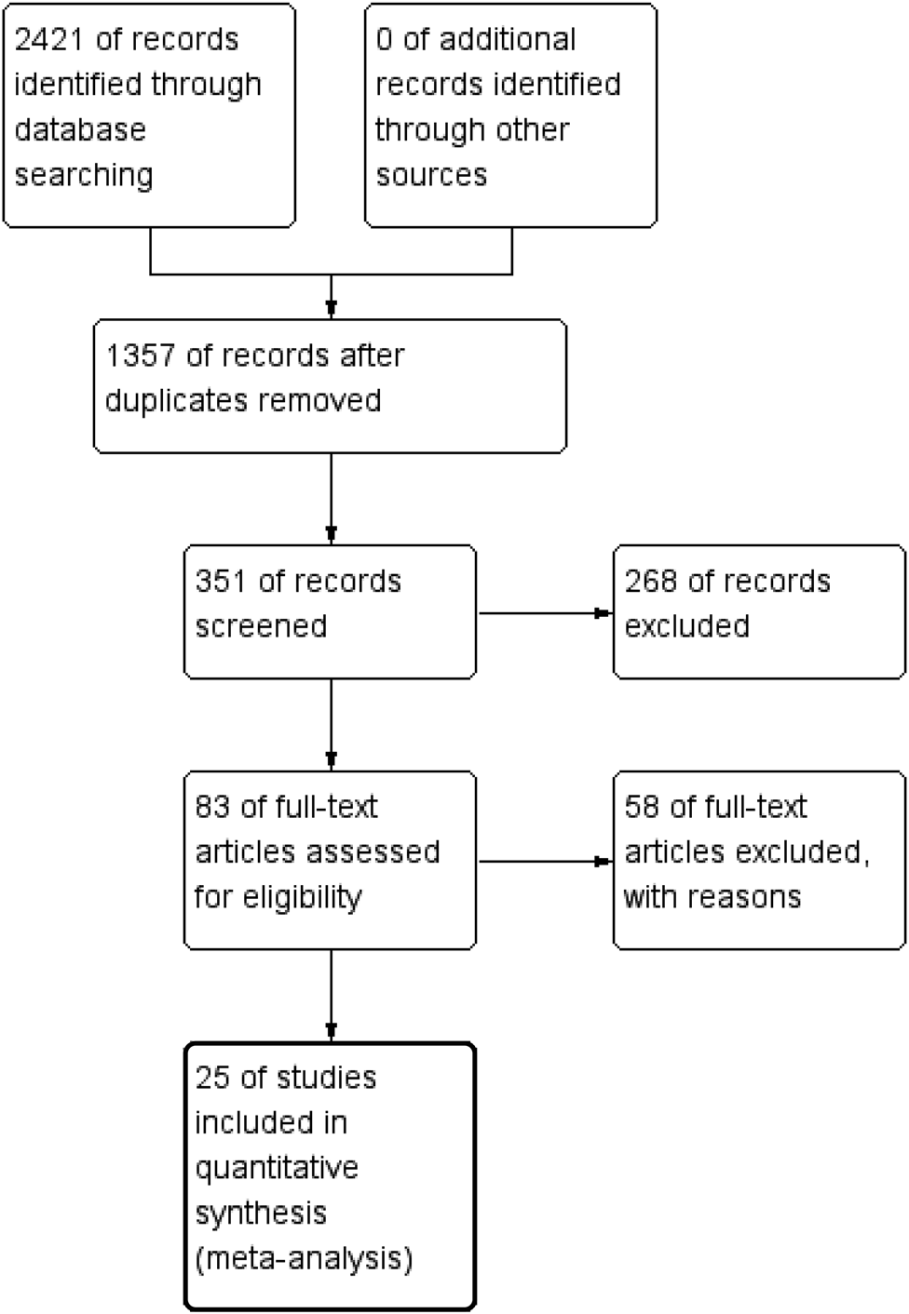
Literature search flowchart.

After searching Chinese and English databases, a total of 1,708 articles related to “the safety and efficacy of different dressings for DFU” were identified. The titles were imported into the NoteExpress V4.X system for de-duplication, resulting in the removal of 1,357 duplicate records and leaving 351 articles. After screening titles and abstracts, 268 studies were excluded due to low relevance or poor quality, leaving 83 articles. Applying the inclusion and exclusion criteria further excluded 58 studies, resulting in a final inclusion of 25 studies in the meta-analysis. The study selection process is illustrated in Figure 1.

### Characteristics of included studies

The 25 studies included in the analysis were all published in English between 2007 and 2025, comprising 50 study arms and a total of 2,421 patients. Among them, 13 studies [9], [11], [12], [13], [14], [15], [16], [17, 19], 20], 23], 25], 28], 29] were randomized controlled trials (RCTs), 6 studies [10], 18], 21], 22], 24], 27], [31], [32], [33] were prospective studies (PS), 1 study [26] was a non-randomized controlled trial (Non-RCT), and 1 study [30] was a retrospective analysis (RA). Detailed characteristics are presented in Table 1.

**Table 1:** Basic characteristics of included studies.

Author	Year	Country	Type	Research group	Control group	Research measure	Control measure	Outcome index
Edmonds et al. [9]	2018	America	RCT	126	114	Sucrose octasulfate dressing	Control dressing	①②
Lázaro-Martínez et al. [10]	2024	America	PS	50	42	Sucrose octasulfate dressing	Conventional dressings	②③
Abdoli et al. [11]	2022	Iran	RCT	30	30	Olive oil dressing+usual care	Usual care	①④
Nasiri et al. [12]	2015	Iran	RCT	15	15	Olive oil dressing+usual care	Usual care	①④⑤⑥
Najafian et al. [13]	2019	America	RCT	20	20	Phytagel+usual care	Usual care+placebo	④⑤⑥
Ghanadian et al. [14]	2024	Iran	RCT	50	44	*Plantago major* extract	Usual care	①⑤⑥
Irani PS et al. [15]	2024	Iran	RCT	33	33	Aloe vera gel	Usual care	①④
Wu et al. [16]	2023	China	RCT	50	50	NPWT	Conventional moist dressings	⑦⑧⑨
Lafontaine et al. [17]	2023	Australia	RCT	63	55	Silver dressings	Non silver dressings	①②
Jude et al. [18]	2007	America	PS	67	67	Silver dressings	CA	②⑤⑩
Salahi et al. [19]	2024	Iran	RCT	25	25	Dermaheal ointment	Placebo	④⑥⑪
Shabib et al. [20]	2024	Thailand	RCT	50	49	Milk ointment	Usual care	①⑥
Henshaw et al. [21]	2014	America	PS	24	84	Propolis	Usual care	①⑥⑦⑨
James et al. [22]	2019	India	PS	27	27	NPWT	Conventional moist dressings	②⑥⑩⑪
Agharazi et al. [23]	2022	India	RCT	38	38	Turmeric ointment	Placebo	①⑥⑩
Campitiello et al. [24]	2021	Italy	PS	29	30	NPWT	Conventional moist dressings	①②⑩
Blume et al. [25]	2008	America	RCT	169	166	NPWT	AMWT	①②⑩⑫
Dong et al. [26]	2017	China	Non RCT	11	11	Oil silver dressing	Silver dressings	⑦⑩
Tajdar et al. [27]	2024	India	PS	25	25	Silver colloid dressing	Conventional dressings	⑥⑩
Bajuri et al. [28]	2024	Malaysia	RCT	20	20	ACC	Silver-based dressing	①②⑥⑩
Probst et al. [29]	2019	Switzerland	RCT	124	124	ACC	Silver-based dressing	①⑥
Xu et al. [30]	2022	China	RA	30	30	Silver foam+ Dermlin dressing	Dermlin dressing	①⑦⑨⑩⑪⑬
Mañas et al. [31]	2025	Spain	PS	31	30	DACC dressing	Usual care	①⑨⑩
Essa et al. [32]	2023	Egypt	PS	40	40	Silver dressings	Conventional dressings	①⑩
Armstrong et al. [33]	2024	America	PS	54	51	PRBM+Standard care	Standard care	①⑥⑩

RCT, randomized controlled study; PS, prospective study; RA, retrospective analysis; NPWT, negative pressure wound therapy; CA, algosteril calcium alginate; AMWT, advanced moist wound therapy; ACC, activated carbon cloth; DACC, dialkylcarbamoyl chloride; PRBM, purified reconstituted bilayer membrane. ① Complete Wound Healing Rate; ② adverse Events; ③ recurrence; ④ wound healing score; ⑤ changes in ulcer depth; ⑥ changes in ulcer area; ⑦ laboratory indicators; ⑧blood perfusion of the affected foot; ⑨ microbial distribution; ⑩ healing time; ⑪ pain score; ⑫ home care duration; ⑬ quality of life.

### Risk of bias assessment of the studies

**Figure 2:**
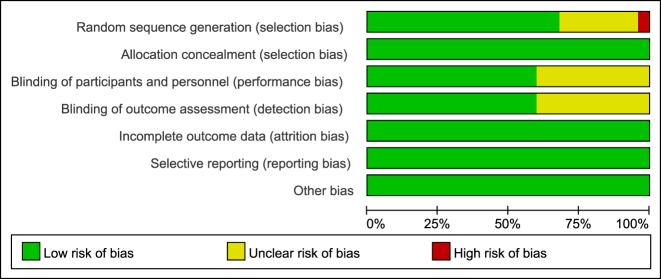
Risk of bias assessment of included studies.

**Figure 3:**
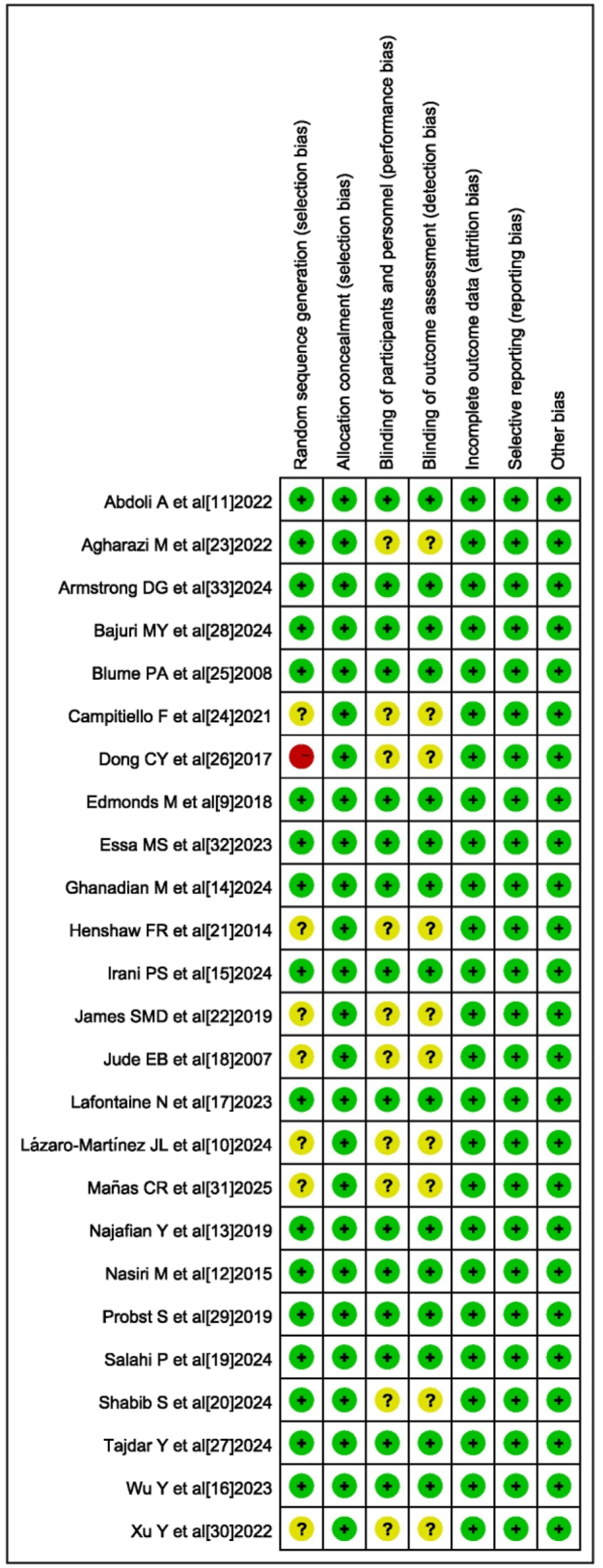
Detailed risk of bias assessment of included studies.

Among the 25 included studies, 17 studies [9], [11], [12], [13], [14], [15], [16], [17, 19], 20], 23], 25], [27], [28], [29, 32], 33] used random sequence generation or random number tables for allocation and were rated as “low risk” for selection bias; 7 studies [10], 18], 21], 22], 24], 30], 31] did not specify the allocation method and were rated as “unclear risk”; 1 study [26] allocated participants based on treatment type and was rated as “high risk”. 15 studies [9], [11], [12], [13], [14], [15], [16], [17, 19], 25], [27], [28], [29, 32], 33] implemented allocation concealment and blinding, and were rated as “low risk” for performance and detection bias. The remaining 10 studies [10], 18], [20], [21], [22], [23], [24, 26], 28], 30], 31] did not describe allocation concealment or blinding and were rated as “unclear risk.” All 25 studies had complete outcome data, showed no selective reporting or other biases, and were rated as “low risk” in these domains. A summary of the risk-of-bias assessment is shown in Figures 2 and 3.

### Meta-analysis of key outcomes

#### Meta-analysis of complete wound healing rate

**Figure 4:**
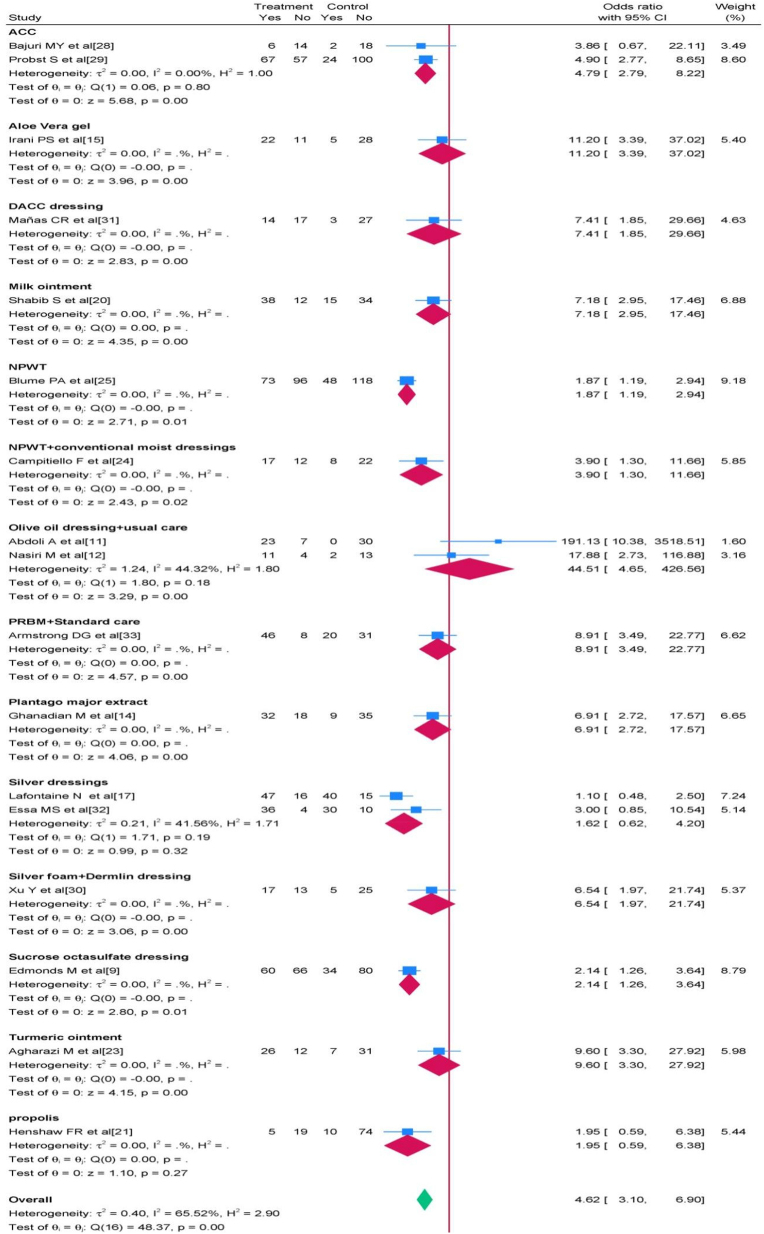
Forest plot of complete wound healing rate.

Seventeen studies [9], 11], 12], 14], 15], 17], 20], 21], [23], [24], [25, [28], [29], [30], [31], [32], [33] reported complete wound healing rates, encompassing 34 study arms and 1,879 patients. Analysis of dichotomous variables revealed significant heterogeneity among studies (p<0.01, I^2^=65.52 %), and a random-effects model was applied. The results showed that the experimental groups had a significantly higher complete wound healing rate compared with the control groups (*OR*=4.62, 95 % CI: 3.10–6.90, *Z*=7.49, p<0.01). In subgroup analyses, the complete healing rates for “Silver dressings” and “propolis” did not differ significantly from the control groups (p>0.05), whereas the remaining 12 subgroups showed significantly higher healing rates than controls (all p<0.05) (Figure 4).

#### Meta-analysis of wound infection

**Figure 5:**
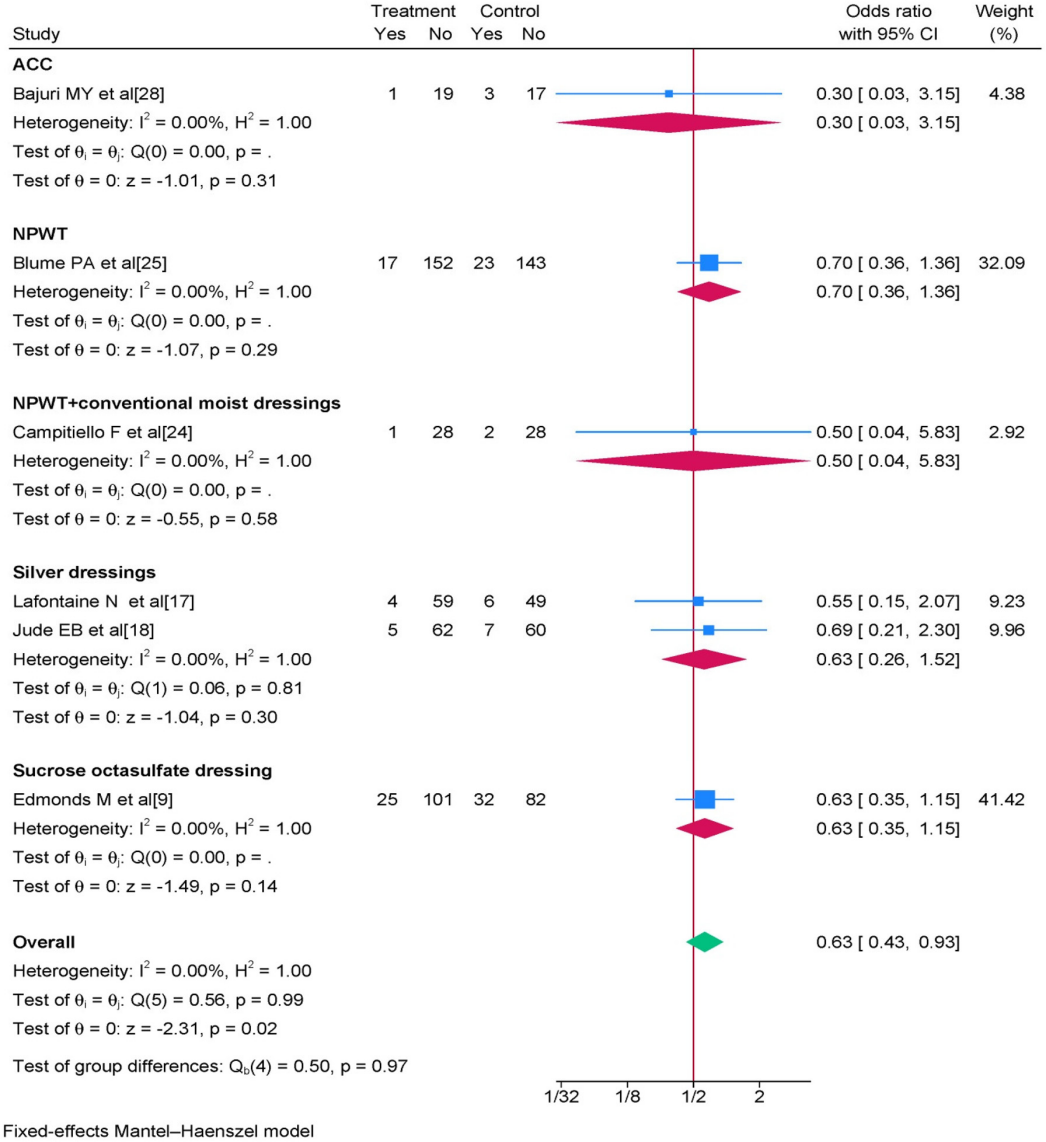
Forest plot of wound infection.

Six studies [9], 17], 18], 24], 25], 28] reported wound infection, including 12 study arms and 926 patients. Analysis of dichotomous variables showed no significant heterogeneity among studies (p=0.99, I^2^=0 %), and a fixed-effect model was applied. The results demonstrated that the experimental groups had a significantly lower incidence of wound infection compared with the control groups (*OR*=0.63, 95 % CI: 0.43–0.93, *Z* = −2.31, p*=*0.02). Subgroup analyses indicated that the incidence of wound infection did not differ significantly from controls in all five subgroups (all p>0.05) (Figure 5).

#### Meta-analysis of amputation rate

**Figure 6:**
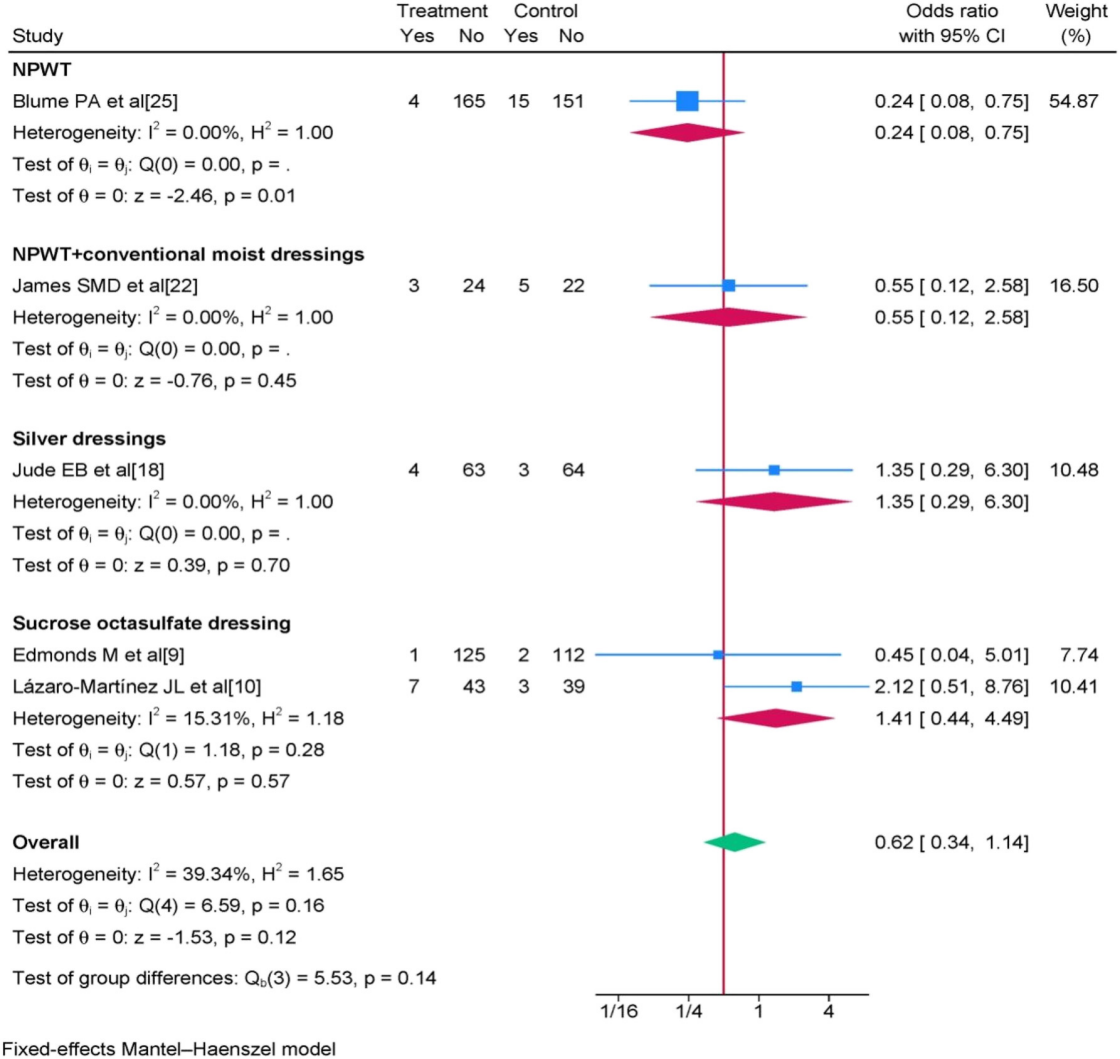
Forest plot of amputation rate.

Five studies [9], 10], 18], 22], 25] reported amputation rates, including 10 study arms and 855 patients. Analysis of dichotomous variables showed no significant heterogeneity among studies (p=0.16, I^2^=39.34 %), and a fixed-effect model was applied. The results indicated no significant difference in amputation rates between the experimental and control groups (*OR*=0.62, 95 % CI: 0.34–1.14, *Z* = −1.53, p*=*0.12). In subgroup analyses, the amputation rate in the “NPWT” subgroup was significantly lower than that in the control group (p<0.05), whereas the remaining three subgroups showed no significant difference compared with controls (all p>0.05) (Figure 6).

#### Meta-analysis of wound healing score

**Figure 7:**
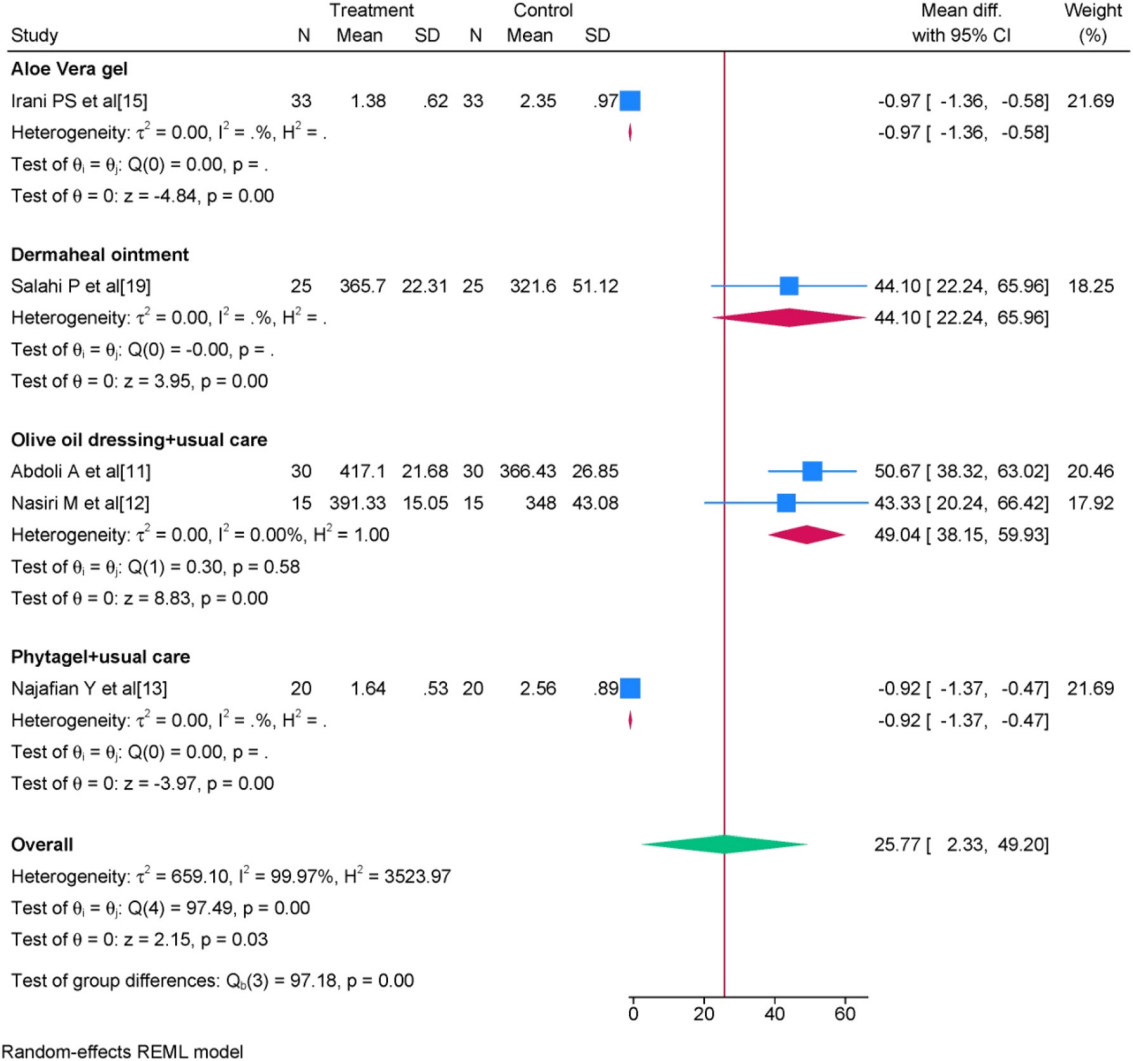
Forest plot of wound healing score.

Five studies [11], [12], [13, 15], 19] reported wound healing scores, encompassing 10 study arms and 246 patients. Analysis of continuous variables revealed significant heterogeneity among studies (p<0.01, I^2^=99.97 %), and a random-effects model was applied. The results showed that the experimental groups had significantly higher wound healing scores compared with the control groups (MD=25.77, 95 % CI: 2.33–49.20, Z=2.15, p=0.03). Subgroup analyses indicated that four subgroups had significantly higher wound healing scores than the controls (all p<0.01) (Figure 7).

#### Meta-analysis of ulcer area Change

**Figure 8:**
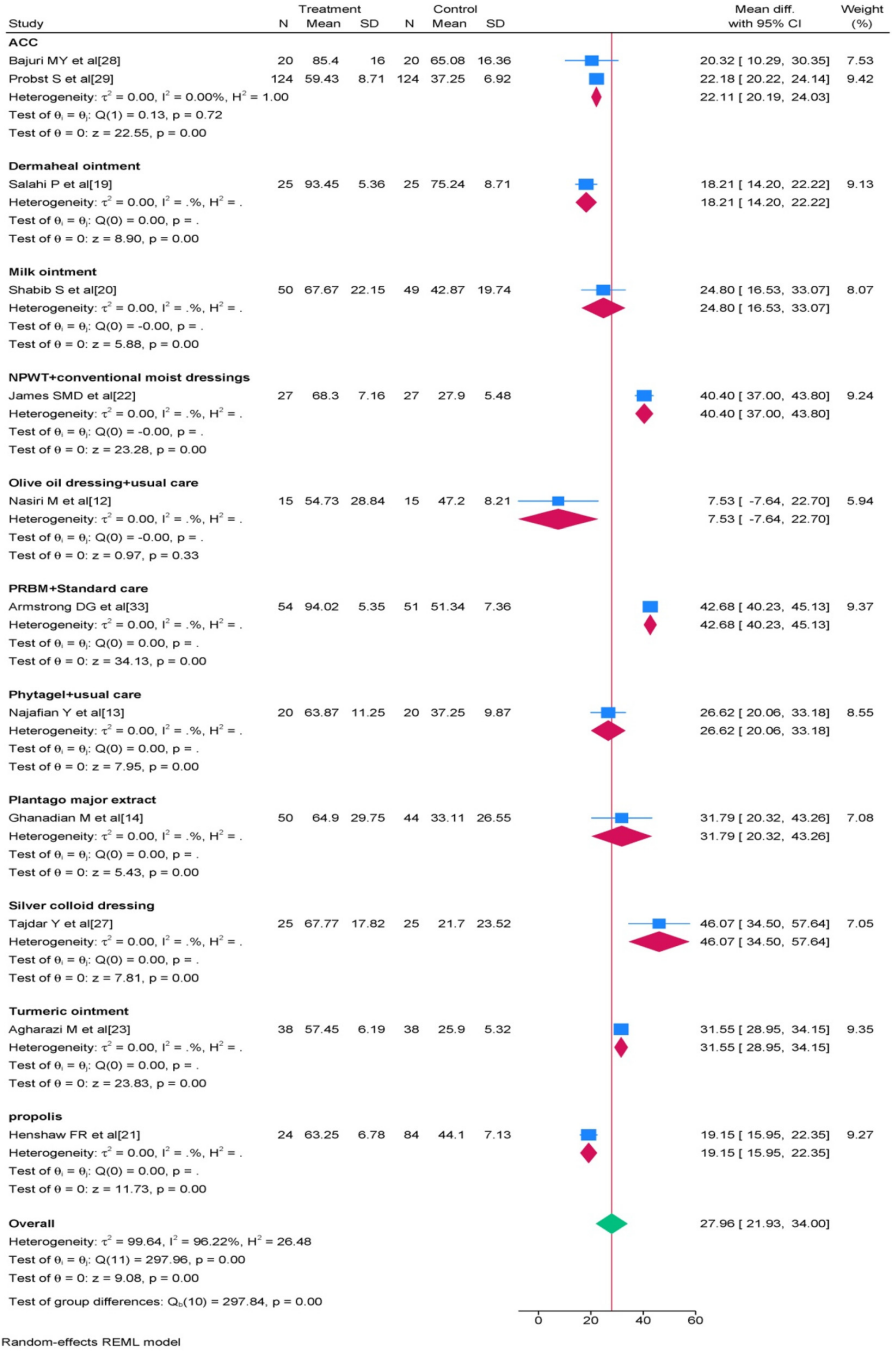
Forest plot of ulcer area change.

Twelve studies [12], [13], [14, [19], [20], [21], [22], [23, [27], [28], [29, 33] reported changes in ulcer area, including 24 study arms and 994 patients. Analysis of continuous variables showed significant heterogeneity among studies (p<0.01, I^2^=96.22 %), and a random-effects model was applied. The results demonstrated that the experimental groups had significantly greater reductions in ulcer area compared with the control groups (MD=27.96, 95 % CI: 21.93–34.00, Z=9.08, p<0.01). Subgroup analyses indicated that the “Olive oil dressing + usual care” subgroup showed no significant difference compared with controls (p>0.05), while the remaining 10 subgroups had significantly greater reductions in ulcer area than controls (all p<0.01) (Figure 8).

#### Meta-analysis of healing time

**Figure 9:**
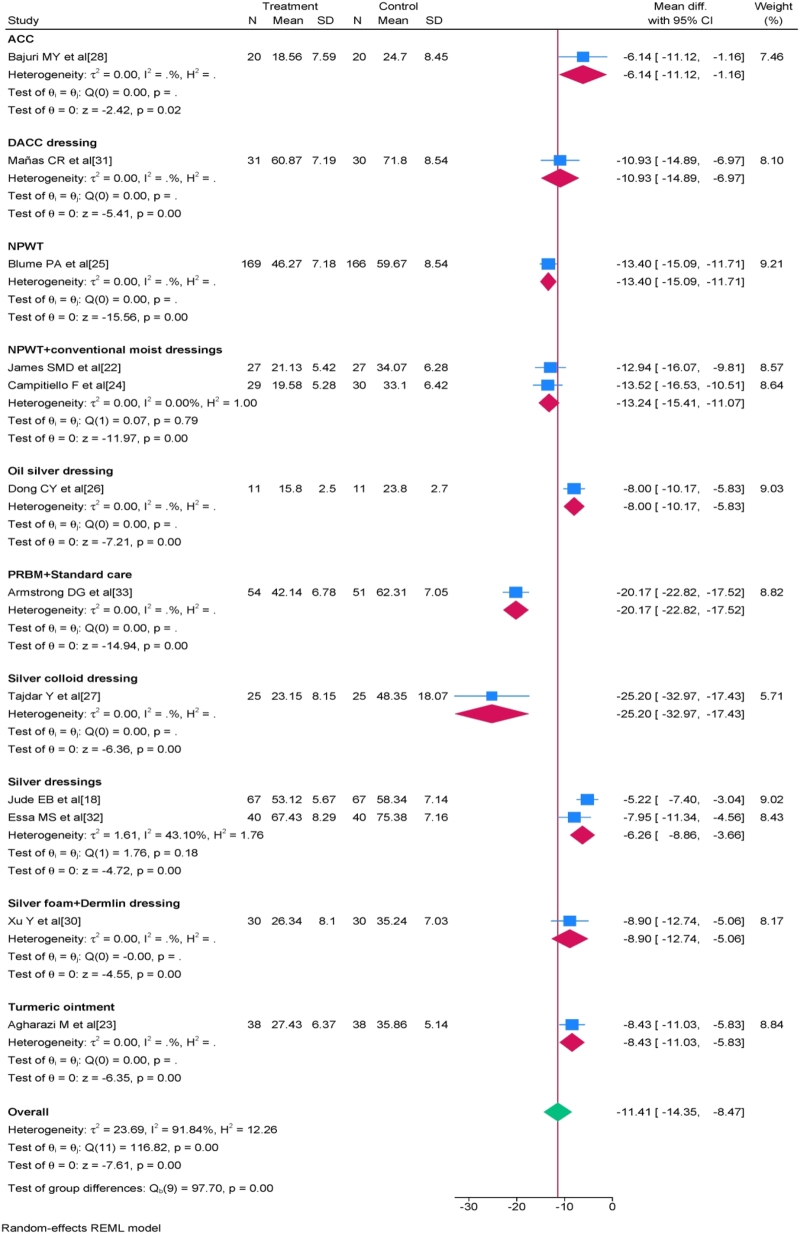
Forest plot of healing time.

Twelve studies [18], [22], [23], [24], [25], [26], [27], [28, [30], [31], [32], [33] reported healing time, including 24 study arms and 1,076 patients. Analysis of continuous variables revealed significant heterogeneity among studies (p<0.01, I^2^=91.84 %), and a random-effects model was applied. The results showed that the experimental groups had significantly shorter healing times compared with the control groups (MD = −11.41, 95 % CI: −14.35 to −8.47, Z = −7.61, p<0.01). Subgroup analyses indicated that all 10 subgroups had significantly shorter healing times than the controls (all p<0.05) (Figure 9).

#### Other outcomes

Due to the limited number of studies reporting outcomes ③, ⑤, ⑦, ⑧, ⑨, ⑪, ⑫, and ⑬, meta-analysis was not performed for these indicators.

### Publication bias analysis

**Figure 10:**
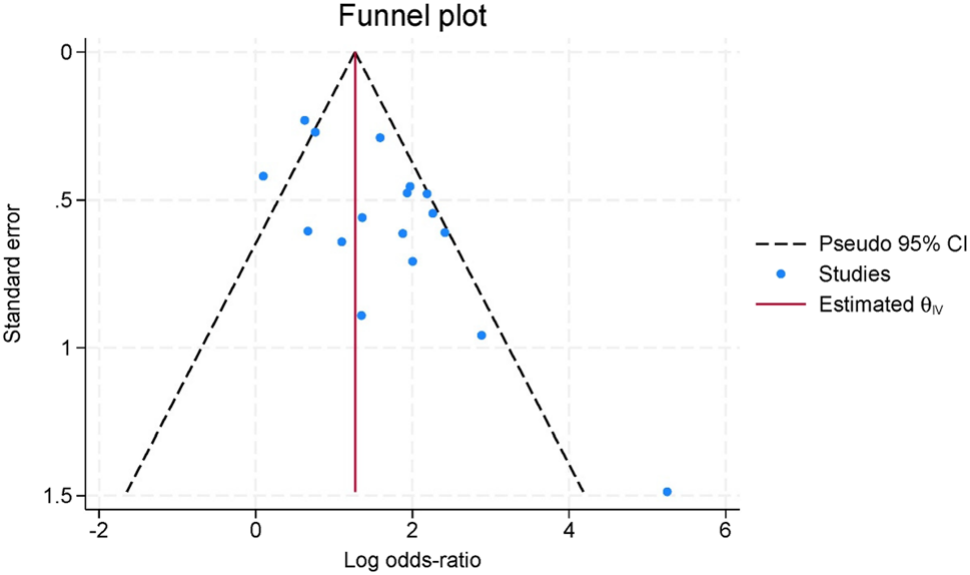
Funnel plot of complete wound healing rate.

Publication bias was assessed for the 17 studies reporting complete wound healing rates. Some studies fell outside the 95 % CI and showed an asymmetrical distribution, suggesting the presence of publication bias, which may be related to study type and intervention methods (Figure 10).

### Heterogeneity analysis

Meta-regression analysis was performed on the 25 included studies, which indicated that publication year (DOR=2.21, 95 % CI: 0.980–1.661), blinding (DOR=2.70, 95 % CI: 0.323–2.024), and outcome measures (DOR=2.60, 95 % CI: 0.224–1.594) were sources of heterogeneity (p<0.05) (Table 2).

**Table 2:** Meta-regression analysis.

Variable	β	SE	p-Value	DOR	95 % CI
Year	0.879	0.399	0.027	2.21	0.980–1.661
Country	0.171	0.088	0.052	1.94	−0.002–0.344
Type	0.031	0.325	0.924	0.10	−0.606–0.668
Blind method	1.173	0.434	0.007	2.70	0.323–2.024
n	−0.022	0.430	0.960	−0.05	−0.865–0.822
Research measure	0.030	0.053	0.564	0.58	−0.073–0.134
Control measure	0.078	0.044	0.072	1.80	−0.007–0.164
Outcome index	0.909	0.350	0.009	2.60	0.224–1.594

## Discussion

### Current research status of DFU

Our study showed that among the 17 studies reporting complete wound healing rates, 12 subgroups – including ACC, Aloe Vera gel, DACC dressing, and NPWT – demonstrated superior healing outcomes compared with controls. Conventional dressings can provide a physical barrier against external bacteria and protect exposed nerve endings, but they generally lack antibacterial activity, often require adjunctive antimicrobial therapy, and have limited absorption capacity for heavily exudative wounds, which can exacerbate deep tissue necrosis [34], 35]. Studies indicate that approximately 19–34 % of patients with diabetes develop DFU, which increases the 5-year mortality rate by 50–68 %, and about 15 % of DFU cases ultimately require amputation [36]. Therefore, controlling the progression of DFU is critical for patients’ physical and mental health. However, once DFU occurs, patients often face challenges such as delayed healing, difficult wound closure, and recurrent infections [37]. In addition, patients frequently lack understanding of the pathogenesis and treatment of chronic wounds, particularly when DFU is accompanied by eczema, cellulitis, or contact dermatitis. Improper care in such cases can directly lead to secondary injury of the affected foot, deep infections, ischemia, and impaired wound healing [1]. In contrast, biologically based wound dressings possess hydrophilicity, biocompatibility, and antimicrobial and anti-inflammatory properties. They offer substantial potential in infection prevention and complex wound adaptation, while providing a warm and moist environment conducive to tissue regeneration [38].

### Advantages and clinical value of different dressings

Supramolecular hydrogels exhibit excellent controllability and can promote angiogenesis, enhance collagen deposition, and reduce wound infection risk and healing time through their intrinsic conductivity, tissue-repair, and antimicrobial properties [39]. ACC contains superabsorbent charcoal components that effectively and continuously absorb tissue exudate, reduce wound odor, and consequently decrease the frequency and duration of dressing changes, while preventing ulcer progression to deeper tissues or osteomyelitis [40]. According to Ontario Health [41], compared with sucrose-free octasulfate dressings, sucrose octasulfate dressings are equally safe and effective, while also reducing the treatment cost of refractory non-infected DFUs and improving patients’ social activity and overall well-being, which aligns with the findings of study [9]. Negative pressure wound therapy (NPWT) can relieve wound pressure, reduce tissue edema, improve local blood flow, and lower the risk of infection [42]. Accordingly, the “NPWT” subgroup showed lower amputation rates than controls. In contrast, the complete wound healing rates in the “Silver dressings” and “propolis” subgroups showed no significant advantage compared with controls.

Study [17] also indicated that, in the treatment of acute DFU without osteomyelitis or tendonitis, silver dressings did not significantly improve wound healing or reduce antibiotic use compared with non-silver dressings. However, Yi et al. [43] reported that in DFU treatment, silver dressings could reduce ulcer area by 27.44 cm^2^ and lower ulcer recurrence by 45 %. Additionally, olive oil, Aloe Vera gel, silver dressings, and silver-impregnated dressings each showed specific advantages in wound healing scores, ulcer area reduction, and healing time. Aloe Vera gel, a natural hydrogel, has demonstrated high therapeutic value in burns, ulcers, and donor site wounds following skin grafting [44]. Silver-impregnated dressings can enhance macrophage antibacterial activity and inhibit bacterial growth [45]. Propolis and aqueous gels have been considered advanced dressings for neuropathic DFU by Monami et al. [46], as they reduce healing time, dressing frequency, and pain compared with conventional dressings or placebo. These findings suggest that different dressings have distinct focal benefits and should be selected according to the primary and secondary needs of DFU patients. It should be noted that due to the limited reporting of outcomes such as pain, quality of life, and recurrence rate in the included studies, meta-analysis and subgroup analysis for these outcomes could not be performed. Future studies should collect as much follow-up data re-examination data as possible to evaluate the long-term efficacy of different dressings.

### Dressing-related factors affecting DFU healing

Funnel plot analysis of the 17 studies reporting complete wound healing rates indicated the presence of publication bias, which may be related to differences in dressing materials. Although modern dressings for DFU each have distinct advantages, pharmacological studies have found that silver sulfadiazine, keratin gels, hydrogels, and aqueous gels exhibit relatively high allergenic potential, whereas non-adhesive dressings, hydrofiber materials, and alginate dressings carry lower risks of hypersensitivity [47]. If patients with DFU are not adequately informed about the properties of dressing materials during treatment, allergic reactions may occur and subsequently impair wound healing. This effect is particularly pronounced in patients with fragile skin barriers, compromised immunity, or poor psychological resilience. Allergic responses triggered by dressing materials or routine diabetes treatments (such as glucose sensors, insulin pumps, or antibiotics) can significantly delay wound healing, increase the incidence of adverse events, and impose a greater caregiving burden [48], 49]. A multicenter study on chronic leg ulcers [50] demonstrated that the number of positive patch test reactions was significantly associated with the duration of ulcerative disease, and up to 59.60 % of patients showed positive patch test reactions to modern dressings. Furthermore, according to the Wagner-Meggitt grading system, DFU is classified from grade 0 to 5, with the appropriate dressing and other treatment strategies varying according to ulcer depth and extent [51]. Therefore, if the selected dressing is insufficient to absorb exudate, control infection, or improve tissue ischemia, it should be replaced immediately, and laboratory indicators and wound healing progress should be closely monitored. In addition, prior to the application of dressings in DFU patients, wound grading and patch testing should be performed. For patients with psychological distress (e.g., excessive anxiety, worry, or nervousness), appropriate reassurance should be provided. Meanwhile, dressing components should be clearly labeled and explained to patients to facilitate the identification of individual-specific allergens, minimize skin irritation, and improve compatibility between the dressing and the wound. These measures may reduce the risk of hypersensitivity reactions and promote wound healing and treatment comfort.

## Conclusions

In summary, compared with conventional dressings, ACC, NPWT, olive oil, hydrogels, and other modern dressings demonstrate favorable safety and clinical efficacy in improving complete wound healing rates, shortening healing time, and reducing infection in DFU patients. However, the specific advantages vary among different dressings, and selection should be tailored to the individual needs of each patient. A limitation of this study is the lack of subgroup analyses stratified by the Wagner-Meggitt classification of DFU, ulcer stage, wound size/depth or infection severity. This deficiency makes it impossible to clarify the precise therapeutic efficacy of different dressings for DFU of specific severity, and the depth of the research remains insufficient. Additionally, heterogeneity remained in the pooled effect estimates due to factors such as publication year, blinding, and outcome measures. Future studies should expand the research scope and further minimize potential biases to improve the reliability of results and effect estimates.
